# Supplementary material for: MetaRibo-Seq measures translation in microbiomes
Source: Nat Commun. 2020 Jun 29;11:3268. doi: 10.1038/s41467-020-17081-z (PMC7324362; doi:10.1038/s41467-020-17081-z)
Supplement: Supplementary file 10 — Supplementary Data 7 [file 41467_2020_17081_MOESM10_ESM.zip › File2/Confidence_High_Taxonomy/298073_out.krona.html]

Javascript must be enabled to view this page.

members
magnitude
magnitudeUnassigned
count
unassigned
taxon
rank

298073\_out

2

2
2
superkingdom

2
1239
phylum

class
186801
1

186802
1
order

species

SRS075773\_contig\_number\_28648
1950886
1


SRS045826\_contig\_number\_contig-100\_62.152733
species
1
1897032
